# From Nanomaterials to Nanofertilizers: Applications, Ecological Risks, and Prospects for Sustainable Agriculture

**DOI:** 10.3390/plants15030415

**Published:** 2026-01-29

**Authors:** Jingyi Zhang, Taiming Zhang, Yukui Rui

**Affiliations:** 1 State Key Laboratory of Nutrient Use and Management, College of Resources and Environmental Sciences, China Agricultural University, Beijing 100193, China; 2 Professor Workstation in Shanghe County, China Agricultural University, Shanghe, Jinan 251600, China; 3 Professor Workstation in Wuqiang County, China Agricultural University, Wuqiang, Hengshui 053300, China

**Keywords:** nanofertilizers, nutrient utilization, soil improvement, environmental safety, soil–microbe interactions

## Abstract

Nanofertilizers have attracted increasing attention as an approach to improve the low nutrient use efficiency of conventional fertilizers, in which only a limited fraction of applied nitrogen, phosphorus, and potassium is ultimately taken up by crops. Beyond their capacity to minimize nutrient losses, nanofertilizers have attracted increasing attention for their possible role in addressing environmental issues, including soil eutrophication and the contamination of groundwater systems. Owing to their nanoscale characteristics, including large specific surface area and enhanced adsorption capacity, these materials enable more precise nutrient delivery to the rhizosphere and sustained release over extended periods, while also influencing soil–plant–microbe interactions. In this review, nanofertilizers are classified into six major categories—macronutrient-based, micronutrient-based, organic, controlled-release, composite, and nano-enhanced formulations—and representative examples and preparation routes are summarized, including green synthesis approaches and conventional chemical methods. The agronomic mechanisms associated with nanofertilizer application are discussed, with emphasis on enhanced nutrient uptake, modification of soil physicochemical properties, and shifts in microbial community composition. Reported studies indicate that nanofertilizers can increase crop yield across different crop species and formulations, while also contributing to improved nutrient cycling. Despite these advantages, several limitations continue to restrict their broader adoption. These include uncertainties regarding long-term environmental behavior, relatively high production costs compared with conventional fertilizers, and the absence of well-defined regulatory and safety assessment frameworks in many regions. Overall, this review highlights both the opportunities and challenges associated with nanofertilizer application and points to the need for further development of cost-effective formulations and standardized evaluation systems that account for their distinct environmental interactions.

## 1. Introduction

In contemporary agricultural systems, fertilizers are essential inputs for preserving soil nutrient status and supporting stable crop yields. However, the effectiveness of conventional fertilizers is severely limited by their low nutrient use efficiency (NUE), a persistent challenge in global agricultural production [1]. A substantial proportion of applied nutrients, including nitrogen, phosphorus, and potassium, is lost through leaching, surface runoff, volatilization, or irreversible fixation in soil. Such nutrient losses lead to higher production costs for farmers and, at the same time, exacerbate multiple environmental impacts, including soil acidification, land degradation, and the eutrophication of surface water bodies. Such issues are particularly pronounced in major agricultural countries, including China, where fertilizer application intensity remains high.

To address these constraints, slow-release and controlled-release fertilizers have been introduced to regulate nutrient release rates and prolong the period of nutrient availability. Although these formulations represent an improvement over conventional fertilizers, their practical performance remains constrained. In many cases, nutrient release rates cannot be adjusted to match crop demand dynamically, and production costs remain relatively high [2]. As a result, these products have not fully satisfied the requirements of sustainable, efficient, and economically viable agricultural systems, leaving scope for further innovation in fertilizer design [3]. Nanofertilizers have recently emerged as a new class of agricultural inputs designed to address these challenges. Constructed using nanomaterial-based carriers and modified through techniques such as microencapsulation and microemulsion processing, nanofertilizers integrate materials such as halloysite nanotubes, chitosan nanoparticles, and nanosilica with coating or encapsulation strategies. By exploiting nanoscale size effects and surface properties, these systems aim to regulate nutrient release, enhance nutrient delivery efficiency, and strengthen interactions among soil, plants, and microorganisms. Owing to these attributes, nanofertilizers have attracted increasing attention as potential alternatives to both conventional and slow-release fertilizers. Despite rapid progress in this field, current research on nanofertilizers remains fragmented. Many studies focus on individual fertilizer types, such as nano-micronutrient formulations or nano-based slow-release systems, and are often conducted under controlled laboratory conditions. Comparatively fewer studies provide integrated analyses that link nanofertilizer classification, synthesis routes (e.g., green synthesis or liquid-phase intercalation), mechanisms of action (including nutrient uptake via apoplastic and symplastic pathways and modification of soil physicochemical properties), field-scale agronomic performance, and potential ecological risks, such as effects on soil microorganisms and soil fauna.

To address these gaps, this review presents a comprehensive overview of nanofertilizers by classifying them into six major categories: nano-macronutrient, nano-micronutrient, nano-organic, nano-slow/controlled-release, nano-composite, and nano-enhanced fertilizers. We summarize their main synthesis strategies, including physical blending, chemical grafting, and biologically mediated green synthesis, and examine their mechanisms of action with particular emphasis on nutrient uptake processes, soil physicochemical modification (e.g., changes in cation exchange capacity and bulk density), and rhizosphere microbial regulation. Furthermore, both the agronomic benefits of nanofertilizers—such as yield improvements in crops including maize and rice—and their potential ecological risks, including nanoparticle accumulation in soil and impacts on soil fauna, are critically evaluated. Finally, current challenges, such as high production costs and underdeveloped regulatory frameworks in China, are discussed alongside future research directions to support the rational and sustainable application of nanofertilizers.

**Figure 1:**
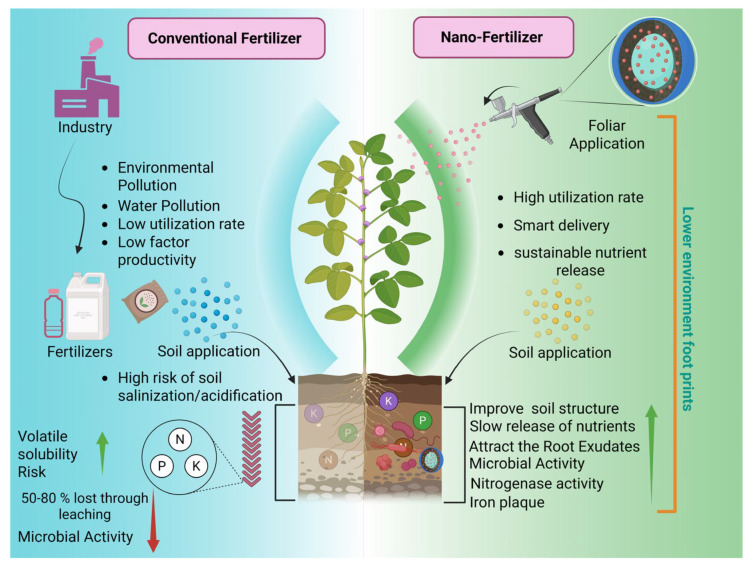
Comparison between conventional fertilizers and nanofertilizers (created with BioRender, https://app.biorender.com/, accessed on 13 January 2026).

For major agricultural countries such as China, where fertilizer costs and environmental sustainability represent key constraints, nanofertilizers may offer practical advantages by reducing material inputs while improving nutrient efficiency. Insights from field-based investigations, including studies conducted under real agricultural conditions, highlight the importance of understanding nanomaterial behavior within fertilizer systems. Such efforts are essential for advancing efficient, environmentally responsible fertilizer strategies and supporting the long-term sustainability of agricultural production (Figure 1).

This study employed a systematic review methodology following the PRISMA (Preferred Reporting Items for Systematic Reviews and Meta-Analyses) guidelines to analyze the existing body of literature concerning preparation methods, mechanisms of action, environmental behavior, and application prospects of nanofertilizers and related nanomaterials in agriculture. Literature searches were conducted using Web of Science and Google Scholar databases. The search strategy utilized specific Boolean combinations of key terms including: “nano” OR “materials” OR “fertilizer” OR “sustained-release” OR “plants” OR “method” OR “toxicity” OR “nutrient utilization” OR “environmental safety” OR “soil” OR “sustainable agriculture” OR “soil–microbe interactions” OR “microorganism” OR “mechanism”.

## 2. Types and Synthesis of Nanomaterials

### 2.1. Definition and Properties of Nanomaterials

Nanomaterials (NMs) are generally described as particles with characteristic sizes in the range of 1–100 nm, positioned between molecules/atoms and macroscopic bulk materials. They mainly consist of two components, namely nanocrystallites and grain boundaries, and exhibit surface and interface phenomena, quantum size confinement, nanoscale dimensional effects, and electron tunneling at the macroscopic level [4]. Changes in nanomaterial size lead to corresponding variations in their physical and chemical properties.

Owing to their reduced particle size, nanomaterials possess a high proportion of surface atoms, which markedly enhances their chemical reactivity. One of the key features of nanomaterials is their high specific surface area combined with increased surface energy, resulting in a large number of reactive sites that underpin their enhanced catalytic efficiency and adsorption capacity [5,6].

### 2.2. Common Types of Nanomaterials

According to their sources, nanomaterials can be categorized into naturally occurring nanomaterials and artificially synthesized nanomaterials. Artificial nanomaterials can be further divided into engineered nanomaterials and secondary nanomaterials [7,8]. Engineered nanomaterials refer to those intentionally designed and fabricated by humans, while secondary nanomaterials are those generated as a result of human activities, such as plastic nanoparticles. Naturally occurring nanomaterials are formed through biogeochemical processes without human influence, such as clay minerals [9], which are naturally sourced nanoscale silicate materials exhibiting special morphologies such as nanorods, nanofibers, nanotubes, nanosheets, and nanoporous structures.

Based on their dimensionality, Nanomaterials are generally categorized into 0D, 1D, 2D, and 3D structures [10]. In 0D nanomaterials, all three spatial dimensions exist within the nanoscale, exemplified by nanoparticles like mesoporous silica nanoparticles, which are commonly employed as nutrient carriers for nanofertilizers. 1D nanomaterials are defined by two nanoscale dimensions, including structures like nanotubes and nanofibers. In contrast, two-dimensional (2D) nanomaterials possess only one dimension at the nanoscale. Representative examples include graphene and various nanosheets. Owing to their large specific surface area and tunable surface chemistry, two-dimensional (2D) nanomaterials, such as graphene oxide, have been widely investigated as nutrient coatings or carrier systems for achieving controlled and sustained nutrient release. In contrast, three-dimensional nanomaterials are composite structures assembled from zero-, one-, or two-dimensional nanoscale building blocks, while the resulting bulk material is not restricted to the nanoscale in any single dimension [11]. The following section summarizes several commonly used approaches for the preparation of nanomaterials [11].

### 2.3. Main Preparation Methods of Nanomaterials

To clarify the preparation strategies of nanomaterials used in nanofertilizers, this section is organized around two main dimensions. The first dimension addresses synthesis approaches, encompassing top-down methods such as physical vapor deposition and mechanical milling, as well as bottom-up chemical routes, including hydrothermal and sol–gel processes [12]. The second dimension addresses environmental compatibility, with an emphasis on green synthesis pathways [13]. Based on this framework, the following sections systematically introduce gas-phase, liquid-phase, and solid-phase preparation methods, with particular attention to those applicable to nanofertilizer development.

#### 2.3.1. Gas-Phase Synthesis Approaches

**Figure 2:**
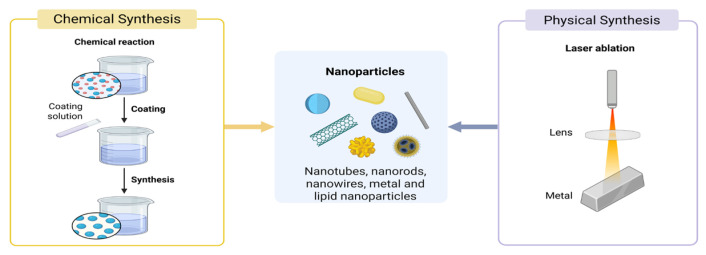
Gas-phase Preparation Method of nanomaterials.

Among gas-phase synthesis methods, chemical vapor deposition (CVD) and physical vapor deposition (PVD) are commonly applied, with CVD representing the more frequently used technique (Figure 2) [14]. In the CVD process, gaseous precursor compounds undergo thermal decomposition or chemical reactions on the surface of a solid substrate under elevated temperatures, typically exceeding 1000 K. By controlling parameters such as temperature, gas flow rate, and substrate type, target products can be fabricated. CVD is commonly applied to the preparation of one-dimensional nanowires, two-dimensional films, and zero-dimensional nanoparticles [14,15]. Li achieved the growth of two-dimensional (2D) layered α-MoO_2_ single crystals through a facile physical vapor deposition (PVD) process [16]. Nano-scale molybdenum trioxide enhances the supplementation of nitrogen-fixing microorganisms by increasing the bioavailability of molybdenum in the rhizosphere soil, thereby promoting biological nitrogen fixation and ultimately improving soybean yield [17].

#### 2.3.2. Liquid-Phase Synthesis Approaches

**Figure 3:**
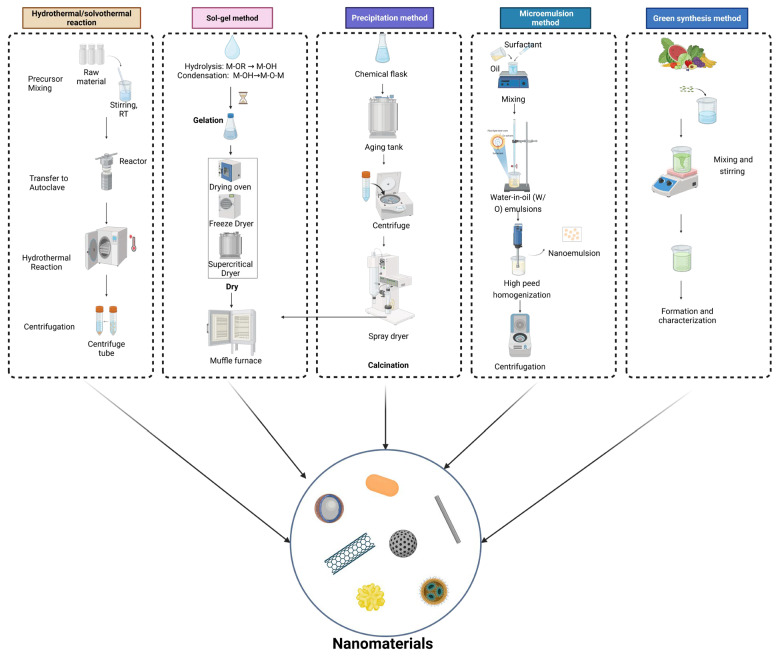
Liquid-phase Preparation Methods of nanomaterials.

The liquid-phase preparation method uses a solution as the reaction medium and produces nanomaterials via chemical reactions or phase transformation processes [18]. The liquid-phase synthesis method employs solutions as reaction media to produce nanomaterials through chemical or phase transformation processes. By controlling reaction conditions, particles with well-defined size and morphology can be obtained. In recent years, green liquid-phase approaches—such as the use of environmentally benign solvents, plant extracts, or mild reaction conditions—have gained increasing attention for their low environmental impact and suitability for sustainable nanofertilizer production (Figure 3) [19].

The hydrothermal/solvothermal method involves the use of a hydrothermal reactor as the reaction vessel, where heating under high temperature creates a high-temperature and high-pressure environment to produce inorganic nanomaterials [20]. Wang et al. explored an efficient and environmentally friendly hydrothermal oxidation process for carbon nanotubes (CNTs) and found that, compared to concentrated HNO_3_ oxidation, hydrothermally oxidized CNTs exhibited superior reinforcement capability for epoxy resins [21].

The sol–gel method, characterized by relatively mild conditions, is one of the earliest techniques for fabricating polymer-based nanocomposites [22]. In early studies, metal alkoxides were first used as precursors for hydrolysis and alcoholysis to form a sol, which subsequently underwent polycondensation to form a gel product. However, it cannot directly produce metallic materials. In addition, sol–gel processing can be carried out under mild conditions, which helps preserve the structural integrity and biological activity of functional components. Owing to these advantages, sol–gel–derived coatings are well-suited for the fabrication of nano-encapsulated fertilizers. Such systems can improve nutrient stability and availability in plant–soil environments [23]. Silica sol and iron-containing silica sol–based suspensions synthesized via the sol–gel method have been applied as pre-sowing treatments for cabbage and cauliflower seeds. These formulations form functional coatings on the seed surface, thereby enhancing seed germination and promoting early-stage plant growth [24].

The precipitation method involves selecting one or more soluble metal salts so that each element exists in ionic or molecular form. Through the use of a precipitant, hydrolysis, evaporation, or sublimation, metal ions are induced to precipitate or crystallize [25,26]. Peng et al. synthesized gelatin/hydroxyapatite (Gel/HAP) nanocomposites via a high-gravity co-precipitation approach, achieving notable protein adsorption capability. Significant improvements in early plant growth, maize yield, rhizosphere microbial activity, and tolerance to NaCl-induced abiotic stress have been achieved through the application of hydroxyapatite nanoparticles assembled with humic substances [27].

The microemulsion method utilizes surfactants to create an anisotropic, thermodynamically stable, and uniformly dispersed mixture of immiscible liquids [28,29]. Variation in the composition of the microemulsion system allows fine control over the morphology and dimensions of the microreactors.

Green synthetic nanofertilizers are produced using environmentally benign approaches in which metal ions are reduced and stabilized by plant-derived extracts to form functional nanomaterials [30]. These plant extracts serve as both reducing and stabilizing agents, allowing the formation of stable nanoparticles or nutrient-loaded nanostructures. The nanomaterials produced through these approaches facilitate regulated nutrient release and assimilation within different plant tissues, thereby enhancing nutrient use efficiency and supporting crop growth and yield improvement [31,32]. For example, zinc oxide nanoparticles synthesized using Catharanthus roseus leaf extract were shown to markedly promote seed germination and early seedling development in Eleusine coracana, with the strongest stimulatory response recorded at a concentration of 500 mg L^−1^ [31]. Violet flower extract–mediated ZnO nanoparticles significantly promoted seed germination, seedling growth, and zinc accumulation in rice at 10 mg L^−1^ [32].

#### 2.3.3. Solid-Phase Synthesis Approaches

The solid-phase synthesis approaches require simple equipment and are easy to operate, but the resulting powders often have low purity and broad particle size distribution, making them more suitable for applications with lower quality requirements [1,17,33,34,35,36,37]. Through a two-step solid-state reaction, Cheng and co-workers developed nanoscale LiFePO_4_ cathode material exhibiting high performance in lithium-ion batteries. Using CH_3_COOLi·2H_2_O, FeC_2_O_4_·2H_2_O, and NH_4_H_2_PO_4_ as feedstock, a LiFePO_4_ precursor was first prepared at room temperature, illustrating a straightforward route for precursor formation, which was then subjected to a high-temperature solid-state reaction to obtain LiFePO_4_ cathode material [38]. A related investigation reported that applying recycled nano-LiFePO_4_ (rn-LiFePO_4_) at rates below 50 mg kg^−1^ produced a range of beneficial effects on peanut growth. These responses were primarily linked to the sustained nutrient release and nanoscale-specific mechanisms of the material, which collectively led to improved photosynthetic capacity, enhanced root system development, and yield gains of 22–34%, accompanied by a notable improvement in seed nutritional quality [39].

The mechanical ball milling method uses equipment such as ball mills and sand mills to physically process powders or dust particles, either refining the material or inducing a reaction, followed by precursor treatment to obtain the target product [40]. It is commonly applied to the preparation of nanopowders. Advantages include moderate cost, scalability for mass production, and environmental friendliness. Zhu et al. prepared nano-MgO using mechanical ball milling, which improved reaction homogeneity and produced nano-MgO particles with a more uniform size distribution of 670 nm [41].

In addition to the mainstream methods described above, there are also more specialized or narrowly applied preparation techniques. Considering their limited universality, methods such as liquid-phase exfoliation and gas-phase condensation will not be discussed in detail here [42,43].

## 3. Application of Nanomaterials in Fertilizer Development and Improvement

The global fertilizer industry is increasingly developing toward slow and controlled-release formulations, high efficiency and precision, resource conservation, and environmental friendliness. Achieving these objectives requires integration with advanced technologies. Within this framework, Zhang Fudao from the Institute of Soil Science and Fertilizer, Chinese Academy of Agricultural Sciences, was the first to introduce the concept of “nanofertilizer.” Nanofertilizer is a branch of nanobiotechnology and refers to a new type of fertilizer constructed using nanomaterial technology and modified through pharmaceutical microcapsule technology and chemical microemulsion technology [34].

### 3.1. Types of Nanofertilizers

#### 3.1.1. Nano-Macronutrient Fertilizers

Nano-macronutrient fertilizers are innovative fertilizers developed using nanotechnology to supply essential macronutrients for plant growth, including nitrogen, phosphorus, and potassium. Calcium phosphate nanoparticles, such as nanocrystalline hydroxyapatite (nAp) and amorphous calcium phosphate (ACP), contain the macronutrients calcium and phosphorus. These nutrients are gradually released as the particles dissolve, which helps reduce nutrient loss caused by soil fixation or runoff [44,45]. They can also be loaded with other macronutrient-containing substances, such as nitrate ions or urea. Compared with conventional fertilizers, nanophosphate formulations can substantially improve phosphorus uptake efficiency while reducing phosphorus losses caused by leaching and surface runoff. As a result, their application contributes to yield improvement while alleviating environmental pressures associated with phosphorus overuse [46]. Nano-scale multi-element fertilizers, designed to enhance the utilization efficiency of macronutrients such as nitrogen, phosphorus, and potassium, are particularly suited to crops with high nutrient requirements, including maize and wheat, as well as to soils that are susceptible to nutrient loss.

#### 3.1.2. Nano-Micronutrient Fertilizers

Nano-micronutrient fertilizers have attracted growing interest in agricultural research as an approach to improve the availability of essential trace elements. By utilizing nanomaterials as carriers or through tailored nanostructural designs, the bioavailability of micronutrients such as iron, zinc, manganese, and molybdenum can be enhanced [47]. These formulations enable localized delivery and regulated release in the rhizosphere or within plant tissues. Although required in relatively small amounts, micronutrients are indispensable for normal plant growth and physiological development.

Among emerging materials, glauconite-derived nanocomposites have been increasingly explored as micronutrient sources for crop fertilization. Previous studies have shown that incorporating copper and boron into glauconite matrices can promote plant development, as evidenced by increases of approximately 7% in seedling length and 6.4% in biomass relative to untreated controls [47]. Halloysite nanotubes, which display a slender tubular shape, were chemically treated by Maximov to load zinc into their multi-scale pores. After application, these nanotubes can attach to leaf surfaces and remain there [48], ensuring a gradual and prolonged release of zinc and associated nutrients. Zinc plays a critical role in plant biochemical and metabolic processes. Sary reported that high doses of nano-micronutrient fertilizers (nano-zinc, nano-manganese, and nano-molybdenum) enhanced vegetative growth, ear characteristics, and yield of maize grown on calcareous soils [49]. Owing to their high bioavailability, nano-based trace element formulations are effective for supplying nutrients that are easily immobilized in soil, such as iron and zinc. These materials are therefore well-suited for calcareous soils and for crops suffering from specific micronutrient deficiencies, for example, iron-deficient lettuce and zinc-deficient maize.

#### 3.1.3. Nano-Organic Fertilizers

Nano-organic fertilizers are novel products formulated on the basis of organic fertilizers, scientifically optimized to satisfy the nutrient demands of diverse crop species, and characterized by “ecological, effective, and broad-spectrum” advantages. Using the “salt-fertilizer pillar support” process, they integrate nanointercalation technology, plant cultivation techniques, chemical engineering, and other disciplines [50,51]. This creates rigid pillars between layers to ensure water and nutrient retention and slow release. Nanointercalation also enhances the ability of layered silicate materials to adsorb and immobilize heavy metals, providing a microenvironment for the growth and reproduction of beneficial microorganisms, continuously increasing soil humus content, and promoting the formation of beneficial microbial communities [52].

Nano-silica fertilizer, an organic-inorganic composite solid fertilizer, holds promise to enhance rice yield while simultaneously improving cost-effectiveness in production and mitigating the risk of ecological pollution [53]. Metal nanoparticles can be integrated with organic components, such as polysaccharides and chitosan, through microbial-mediated green synthesis to form nano-biocomposite fertilizers (NBFs), offering an alternative approach to overcoming several limitations associated with conventional fertilizers [54]. Nano-organic formulations exhibit a pronounced ability to retain both water and nutrients, which contributes to improvements in soil structural integrity and overall stability. Owing to these functional attributes, such materials are especially well-suited for application in ecological farming systems and show considerable potential for the remediation of degraded soils, including those impacted by heavy metal contamination.

#### 3.1.4. Nano Slow/Controlled Release Fertilizers

Owing to their small particle size, elevated surface-to-volume ratio, and enhanced interfacial interactions, these materials facilitate gradual nutrient release and allow release rates to be more closely aligned with crop nutrient demand [55]. These fertilizers have high utilization efficiency, reduce environmental risks from nutrient loss, and improve crop productivity. Common types, classified by dissolution properties, include sulfur-coated slow-release fertilizers, polymer-coated fertilizers, bio-composite-coated slow-release fertilizers, and chemical/physical degradable slow-release fertilizers [56,57]. Physical blending, chemical grafting, and impregnation adsorption are commonly used techniques for modifying slow/controlled release fertilizers with nanomaterials.

Zhou et al. developed a fertilizer that enhances plant growth while providing both slow nutrient release and water-retention capacity, using a strategy that combined liquid-phase intercalation with cross-linked gel formation [58]. The introduction of sodium alginate reinforced the framework’s mechanical strength, while modulating its pore structure to enhance water absorption and nutrient release kinetics. This dual-action formulation was shown to stimulate substantial growth in both crop foliage and root systems [58]. Maxim et al. developed a multifunctional controlled-release nano-composite fertilizer composed of glauconite and nitrogen nutrients in a 1:1 mechanically activated mixture, which reduced nutrient leaching rates compared with urea and slowed nutrient release [59]. Results from field trials showed that nano urea performed better than conventional urea in rice cultivation. Its application led to higher grain yield and greater nitrogen accumulation in grains and straw, together with a significant increase in total nitrogen content [60]. By regulating nutrient release rates, nano slow-release systems help reduce environmental risks associated with fertilization. Such systems are particularly appropriate for crops with long growth periods, including fruit trees and cotton, as well as for dry or highly permeable soils [12]. Wheat biochar (WBC) and wheat nano-biochar (WBNC) were prepared at two pyrolysis temperatures and subsequently loaded with nutrients by impregnation. Among the tested materials, WBNC produced at 350 °C showed an optimal pH of approximately 7.22 and a well-developed porous structure, as confirmed by SEM analysis. Its application significantly improved soil fertility, with multiple soil parameters and properties showing positive responses [61].

#### 3.1.5. Nano-Composite Fertilizers

Nano-composite fertilizers are produced by integrating nanocomposite materials with conventional fertilizer nutrients and are most commonly applied in controlled-release systems [62]. Among these, chitosan-based nano-composite fertilizers have been widely studied. Chitosan, a biopolymer derived from chitin in shrimp exoskeletons, can function as an effective carrier for nutrients such as proteins and humic acids, thereby facilitating nutrient delivery to plants [62]. Previous work presented at the IEEE 2nd International Conference on Advances in Electrical, Electronics, Information, Communication, and Bio-Informatics in 2016 suggested that the use of such fertilizers could reduce application rates and frequency, improve water-use efficiency, enhance cost-effectiveness, and provide additional photoprotective benefits.

Further improvements in nutrient release performance have been achieved through material modification. For instance, embedding montmorillonite (MMT) into chitosan hydrogels substantially enhanced nutrient liberation, with phosphorus release increasing from 22.0% to 94.9% and potassium release from 9.6% to 31.4% [63]. As a result, chitosan–MMT nanocomposite hydrogels exhibited effective control over fertilizer release under soil conditions. Soil pH is a key factor governing nutrient bioavailability. In this context, Shakoor et al. applied sulfur-rich organic phosphate and bio-organic phosphate composite materials in maize cultivation and observed a reduction in soil pH under calcareous conditions, which in turn improved nutrient availability and promoted plant growth [64]. Owing to their combined advantages of relatively low cost, high nutrient content, and favorable agronomic performance, nano-composite fertilizers can supply balanced nutrition while simultaneously improving soil properties. These formulations are therefore particularly suitable for nutrient-deficient or calcareous soils, as well as for crops with high and diverse nutrient requirements.

#### 3.1.6. Nano-Enhanced Fertilizers

Nano-enhanced fertilizers are novel nanofertilizers that fully exploit the surface and nanoscale effects of nanomaterials, combined with macro- and micronutrients required by plants [65]. They improve the uptake of nutrients by plants, while simultaneously reducing fertilizer losses and fixation processes in soils, thereby increasing overall fertilizer use efficiency. In addition, they promote plant growth and development, which ultimately contributes to higher crop yields [66]. With a primary focus on enhancing nutrient uptake and yield performance, nano-enhanced fertilizers can be applied across a wide range of soil types and are suitable for economically important crops, including lettuce and wheat. However, the selection of functional enhancers should be tailored to specific crop requirements.

Higher dosages of nano-chelated iron drive substantial increases in total protein, soluble sugar, and free amino acid content in lettuce plants, activating metabolic pathways to enhance primary and secondary metabolite biosynthesis while efficiently mobilizing iron and micronutrients for plant utilization [67]. Yang et al., in greenhouse pot and field trials, tested seven different nanofertilizer enhancers on wheat and observed significant improvements in nitrogen accumulation and nitrogen use efficiency (by 4–16% and 10–20%, respectively), as well as upregulation of genes associated with nitrogen uptake and metabolic processes [68].

**Figure 4:**
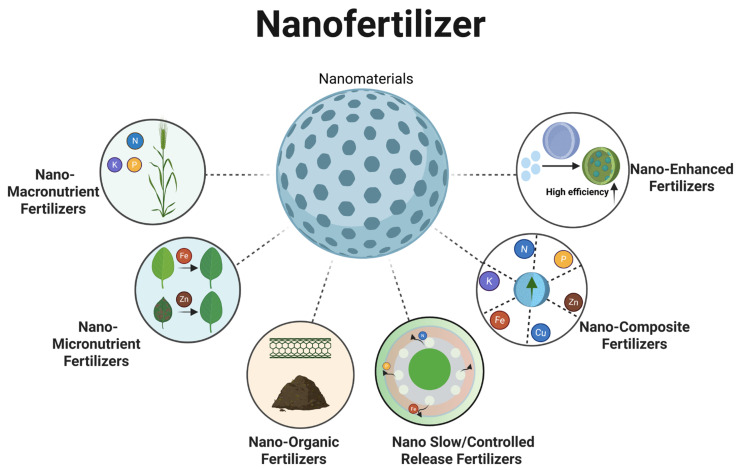
Types of nanofertilizer.

Overall, controlled-release performance is more pronounced in slow-release and composite systems, whereas organic and composite formulations better support soil improvement. Targeted supplementation with macro- or trace-element formulations should be guided by crop demand and soil conditions (Figure 4).

### 3.2. Mechanisms of Nanomaterials in Fertilizer Applications

The introduction of nanofertilizers has been reported to enhance seed germination performance, potentially by increasing internal seed temperature, modifying water distribution within seeds, or activating specific bioactive properties [69]. In modern agricultural systems, nanofertilizers may contribute to meeting crop nutrient demands through a dual mechanism. On the one hand, their small particle size (1–100 nm), which is comparable to or smaller than the pore size of plant cell walls (30–50 nm), may allow them to penetrate cells more readily and facilitate nutrient delivery to the plasma membrane. On the other hand, nanofertilizers possess a specific surface area that can be up to 100 times greater than that of conventional fertilizers, which may promote closer interaction with plant surfaces and improve nutrient uptake efficiency [70].

At the nanoscale, materials may also be capable of crossing plant cell barriers and modifying the rhizosphere environment, for example, by regulating soil pH or influencing soil aeration, thereby potentially facilitating nutrient acquisition by plants [44]. Moreover, certain nanomaterials can function as elemental fertilizers, as they may be absorbed and utilized by plants with limited residual environmental accumulation. By modulating a range of cellular and metabolic processes—including growth-related activities such as cell division and photosynthesis, as well as stress-associated pathways involving vacuolar sequestration, hormonal signaling, calcium signaling, and secondary metabolism—nanofertilizers are considered to have the potential to enhance overall plant vigor and stress resilience under specific conditions [71,72]. A wide range of nanomaterials, including carbon nanotubes, metallic nanoparticles, and metal oxides, have been shown to be taken up by plants through roots, leaves, and other organs, a process that appears to be closely related to their chemical properties. In one study, Badawy et al. reported that the combined application of nano-silicon and nano-selenium fertilizers to rice promoted plant growth, increased grain yield, and alleviated the adverse effects of salinity stress [73]. Similarly, in maize, Elsabagh found that nano-structured water treatment residues used as fertilizer significantly enhanced plant growth and increased phosphorus content compared with conventional fertilizer treatments [74].

**Figure 5:**
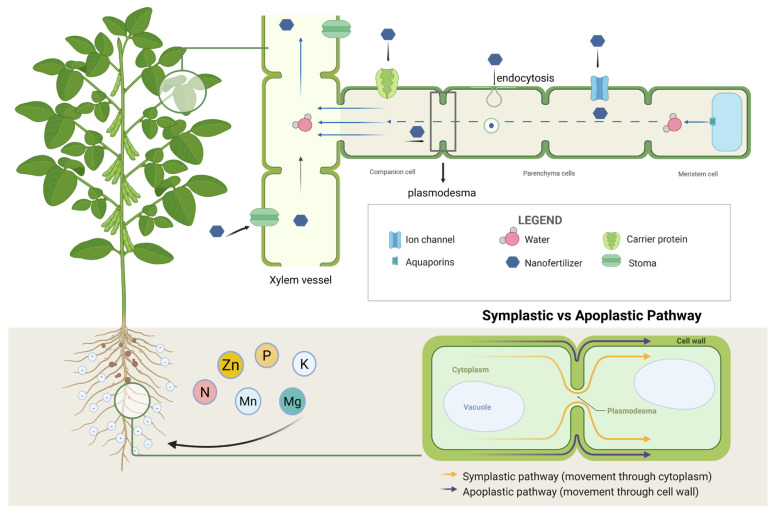
The transport mechanism of nano fertilizers in plants.

Nanomaterials can enter plants and be transported between cells and tissues (Figure 5). The root surface is relatively rough and typically carries a negative charge due to the secretion of mucilage or low-molecular-weight organic acids by root hairs, which may facilitate the accumulation of positively charged nanoparticles on the root surface [75]. In plant roots, nanomaterials may be absorbed through both apoplastic and symplastic pathways, and they can also enter leaves via stomata [76]. Using transmission electron microscopy, Huang observed that copper oxide nanoparticles migrated between cells through both symplastic and apoplastic routes [77]. Nanomaterials may cross plant cell membranes through carrier proteins, aquaporins, ion channels, or endocytosis [78]. Once absorbed, they are transported within plants through vascular tissues, moving toward leaves via the xylem and toward roots via the phloem [79], and may also be transferred between adjacent cells through plasmodesmata [48]. During the first 24 h, intact nano-hydroxyapatite (nHAP) adhered to the root epidermis without detectable dissolution. Over the subsequent 24 h, nHAP penetrated the cell wall barriers of mature epidermal and cortical cells, where it dissolved in the acidic microenvironment of the cell wall matrix [76]. Nano-LiFePO_4_ (n-LiFePO_4_) derived from recycled batteries enhanced maize–peanut intercropping performance by stimulating root growth and rhizosphere processes. It increased root biomass, promoted root exudation, and facilitated phosphorus and iron uptake. These effects were linked to higher acid phosphatase activity, improved phosphorus solubility, and activation of iron-cycling bacteria in soil [80].

**Figure 6:**
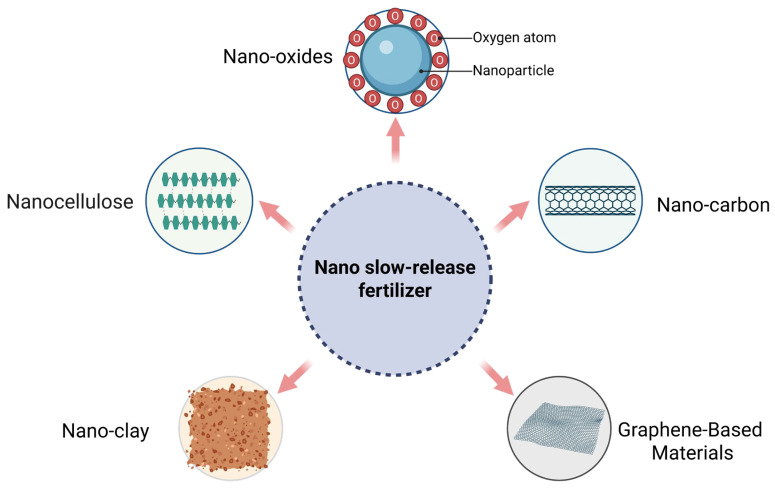
Nano materials applied in slow-release fertilizers.

Nanomaterials such as nano-oxides, nanocellulose, nanocarbon, and nanoclays, when used as fertilizer carriers or fillers, can enable more gradual and controlled nutrient delivery (Figure 6). However, the extent of nutrient release is strongly influenced by local conditions, including temperature, soil moisture, pH, biological processes, and soil type [2,81]. In nano-controlled-release fertilizers, coated fertilizers utilize the encapsulation structure of nanomaterials to achieve controlled nutrient release. Physical blending, chemical grafting, and impregnation-adsorption are the primary techniques used to modify slow-release fertilizers with nanomaterials [44]. In addition to single nanomaterials, nano-composites are also prepared for use in controlled-release fertilizers. These composites integrate multiple nanomaterial components and are regarded as an optimization over single-material nano-controlled-release fertilizers.

Nanomaterials can also indirectly improve fertilizer efficiency by regulating the soil microenvironment. Specifically, they can adsorb heavy metal ions and harmful organic substances in the soil, thereby improving soil conditions [82]. For example, nano-phosphate fertilizers may immobilize these harmful components in agricultural soils, preventing their translocation to edible plant tissues [83]. Moreover, nanomaterials can create favorable microhabitats that facilitate the establishment and activity of beneficial soil microorganisms, including nitrogen-fixing bacteria and rhizobia, thereby supporting their growth and proliferation [62,63]. The resulting increase in microbial activity enhances soil nutrient transformation processes, allowing plants to access a greater proportion of available nutrients and supporting more vigorous growth. Experimental evidence further supports these effects. The application of graphite nano additive (GNA) at concentrations of ≤100 mg kg^−1^ soil increased soil microbial biomass carbon by the third week and significantly enhanced plant yield by the seventh week. Rhizosphere enzyme activities were also markedly elevated in the fifth week, coinciding with the peak growth stage of lettuce [84]. Field experiments have further demonstrated the practical applicability of nano urea in rice production. Compared with conventional urea, nano urea significantly enhanced grain yield and nitrogen accumulation in both grains and straw, as well as overall nitrogen content [85].

## 4. Effects of Nanofertilizers on Soil Ecosystem

### 4.1. Effects on Soil Physicochemical Properties

Building on the interactions between nanomaterials and soil biological processes described above, nanofertilizers can also influence soil physicochemical properties. Different types of nano-organic fertilizers can, to varying degrees, affect these properties. In southern and central Africa, a simple ball-milling-based nanotechnology has been developed to obtain nanoscale pyrite for fertilizer application [82]. This mineral provides a “nitrogen–phosphorus–potassium equivalent” nutritional effect in rice, thereby reducing the need for conventional NPK fertilizers, mitigating soil pollution, maintaining soil health, and promoting sustainable land use. In addition, nano-organic fertilizers have been shown to reduce the bulk density of the tilled soil layer, enhance soil aggregate stability, and increase overall soil porosity [86].

At the same time, nanomaterials can modify soil physical properties by improving water-holding capacity, increasing Atterberg limits, and promoting the development of aggregated soil structures. Through the formation of porous and stable three-dimensional networks, these materials enhance the retention of both water and nutrients within the soil matrix [87]. Microscopic observations provide direct evidence for these effects. Using scanning electron microscopy and related imaging techniques, Guo et al. observed that soil particles tended to aggregate around nanosilica particles, leading to localized changes in particle dispersion and spatial arrangement. This aggregation increased the proportion of pores larger than the nanoscale, thereby improving soil aeration and water permeability [88]. In addition, nanosilica particles can form stable aggregates that expand soil pore space, improve root penetration and expansion, and ultimately create a more favorable physical environment for plant growth [86].

Zeolite/hydroxyapatite hybrid nanocomposites (ZHNCs) represent another effective class of nano-composite fertilizers for soil improvement. Their application has been reported to increase cation exchange capacity, reduce soil bulk density, enhance porosity, and maintain suitable soil moisture conditions [87]. By supporting the retention of organic nutrients and improving overall soil quality, these materials further contribute to enhanced plant growth and soil fertility.

### 4.2. Effects on the Soil Biological Environment

**Figure 7:**
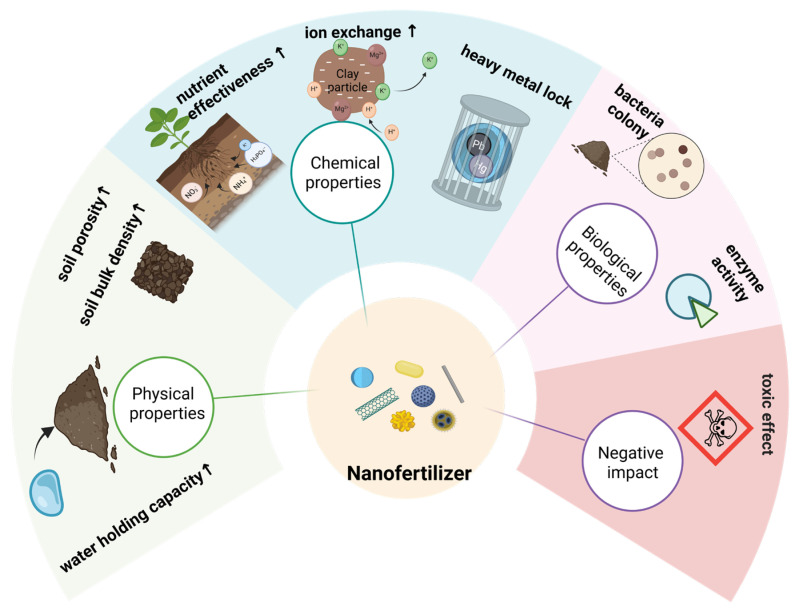
Impacts of Nanofertilizers on Soil Properties & Risks.

Nanomaterials have been reported to improve the soil microecological environment by promoting the activity and proliferation of beneficial microorganisms, thereby facilitating nutrient turnover processes (Figure 7) [89]. In the context of wheat cultivation, the application of nano-silicon fertilizers was found to positively influence soil bacterial community structure, leading to increases in bacterial richness, diversity, and evenness. At harvest, the Chao1, Shannon, and Pielou’s indices were 23.47%, 4.91%, and 3.28% greater, respectively, in treated plots than in controls, with statistically significant differences (*p* < 0.05) [90].

Field-based studies have shown that two applications of nano-selenium fertilizer at the potato seedling stage can markedly attenuate the severity of black spot disease. This protective effect was accompanied by broader improvements in agronomic performance, including yield enhancement and significant gains in tuber quality attributes such as dry matter content, vitamin C concentration, crude protein levels, and selenium enrichment. At the physiological level, nano-selenium treatment was associated with a pronounced upregulation of antioxidant defense systems, as evidenced by increased activities of glutathione peroxidase, peroxidase, polyphenol oxidase, superoxide dismutase, and phenylalanine ammonia lyase [91]. Complementary evidence reported by Upadhyay further supports the agronomic potential of nanofertilizers in nutrient management strategies. Specifically, foliar application regimes supplying 75% of the conventional nitrogen input via nanofertilizers were shown to represent a sustainable approach for enhancing crop growth, yield formation, and rhizosphere biological activity. Notably, two foliar applications of nano-nitrogen, applied either independently or in combination with nano-zinc, reduced overall nitrogen requirements by approximately 25% while simultaneously fostering greater microbial diversity and improved structural stability within soil microbial communities [89,92].

The application of nano slow- and controlled-release fertilizers can increase soil nutrient content, enhance soil enzyme activity, and promote microbial growth, thereby effectively reducing nutrient loss, optimizing soil ecosystem health, and offering significant application potential. Teng et al. reported that the application of controlled-release nanofertilizers resulted in marked enhancements in soil biochemical activity and microbial abundance. Relative to untreated soils, dehydrogenase activity increased by 37.4% and catalase activity by 21.3%, while the populations of bacteria, actinomycetes, and fungi rose by 50%, 72%, and 208%, respectively [89]. At the same time, the cytotoxicity of certain nanoparticles toward bacteria has been attributed to the release of toxic components, such as heavy metals or their ionic forms. Semiconductor nanocrystals, often known as quantum dots (QDs), are generally fabricated using transition metals or noble metals as their core components. These cores are often encapsulated by inorganic shells, including ZnS or CdS, and further stabilized using organic ligands [93].

Although nanofertilizers represent a promising strategy for improving nutrient use efficiency in agricultural systems, their potential adverse effects on soil biotic communities warrant careful consideration [94]. Owing to their pronounced surface reactivity and elevated specific surface area, nanoparticles have been shown to adversely affect soil microbial communities. These effects may manifest as shifts in microbial community composition, declines in microbial diversity, and disruptions of key soil ecological processes. Shah et al. observed that nano-zinc oxide fertilizer increased the fraction of unstable zinc within microbial biomass and consequently lowered bacterial and fungal colony-forming units, indicating adverse effects on microbial abundance and activity [95]. Nanoparticles may also cause physiological damage to soil invertebrates (e.g., nematodes, springtails, earthworms) through direct contact or ingestion, affecting their growth, reproduction, and behavioral patterns [96]. In an experiment by Mohammed, earthworms were maintained in soils containing either balanced NPK compound fertilizers or balanced NPK nano-composite fertilizers. Results showed that earthworm body weight and relative growth rate decreased with increasing fertilizer concentration [92], indicating that nanofertilizers can adversely affect the normal growth and development of soil fauna.

Rashid et al. found that incorporating nano-biochar into fertilizers, even at low levels, supported microbial community functions, improved nutrient cycling, and enhanced nutrient uptake in maize [97]. However, the highest concentration of nano-biochar significantly reduced microbial biomass nitrogen and carbon, soil potassium, and maize potassium uptake, causing toxicity to soil microorganisms and related ecosystem services [97].

Direct soil application of silver nanoparticles (Ag NPs) has been shown to enhance microbial metabolic activity and increase the relative abundance of Proteobacteria and Acidobacteria. However, elevated concentrations exhibit detrimental effects on microbial cells: Ag NPs substantially reduce the copy number of amoA genes (encoding ammonia monooxygenase) in soil bacteria and archaea, while drastically altering the compositional structure of fungal communities [98].

**Figure 8:**
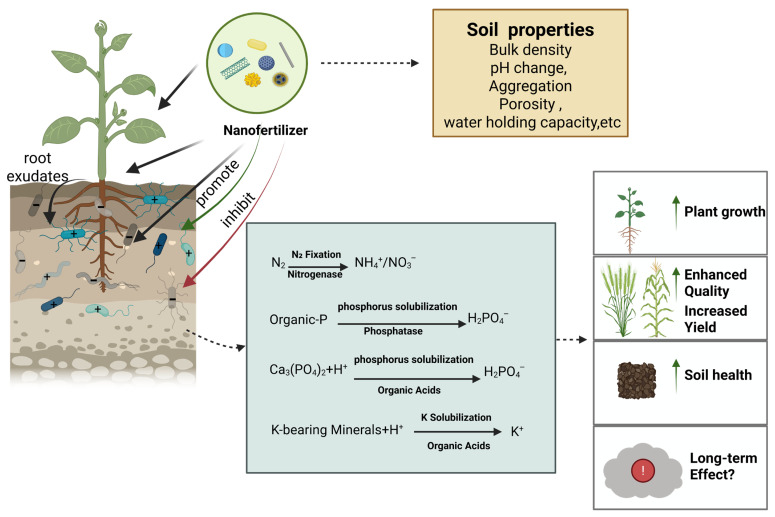
Nano fertilizer-rhizosphere microorganisms-nutrient cycle.

Collectively, these findings indicate that variations in nanofertilizer application rates can influence both the composition and functional activity of soil microbial communities. This effect arises because concentration gradients directly regulate microbial growth and metabolic processes, thereby shaping the ecological functions in which these communities are involved (Figure 8). Consequently, careful control of application dosage is necessary when designing nanofertilizer strategies to preserve soil ecological balance.

Beyond dosage effects, the migration and transformation of nanoparticles within soil systems, together with their interactions with coexisting contaminants, may further enhance nanoparticle bioavailability and amplify potential ecological risks. Such processes can generate secondary effects that extend to soil biogeochemical cycling. For example, Huang et al. reported that copper oxide–based nanofertilizers induced significant phytotoxicity in plant root systems when applied at higher concentrations [77]. Furthermore, CuO nanoparticles modified with trisodium citrate (TC-CuO NPs) exhibited increased translocation efficiency through molecular-level regulatory mechanisms, leading to enhanced accumulation in aboveground plant tissues. These observations suggest that nanofertilizers are capable of migrating and transforming within plant–soil systems by modulating plant gene expression and altering local soil environmental conditions.

Taken together, available evidence supports a concentration-dependent and type-specific framework for assessing the impacts of nanofertilizers on soil ecosystems [93]. When environmentally benign nanofertilizers are applied at appropriately optimized low doses, they primarily improve soil physicochemical properties, stimulate beneficial microbial activity, enhance nutrient cycling, and support crop productivity [97]. By contrast, adverse ecological effects—including microbial community disturbance, impairment of soil fauna, phytotoxicity, and disruption of nutrient cycling—are predominantly associated with excessive application rates of certain nanoparticle types [77]. Overall, the combined consideration of application dosage and material type is essential for maximizing agronomic benefits while minimizing ecological risks, and constitutes a key principle for the sustainable deployment of nanofertilizers (Table 1).

## 5. Challenges and Prospects of Nanomaterials in Fertilizer Applications

### 5.1. Challenges in Nanomaterial-Based Fertilizer Applications

Further investigation is required to better understand the long-term effects and environmental safety of nanomaterials. To address existing regulatory gaps, two critical issues remain to be clarified [101]. As an initial step, nano-specific risks requiring targeted consideration should be explicitly identified, particularly those related to the environmental fate and behavior of nanomaterials—such as their migration and transformation within soil–plant systems, as well as their potential for bioaccumulation and trophic transfer along food chains [102]. Second, regulatory frameworks governing nanofertilizers require further development, particularly with respect to risk assessment and life cycle assessment. These evaluations should extend across the entire life cycle of nanofertilizers, from material synthesis and agricultural application to environmental degradation and end-of-life behavior [103]. Although numerous studies have demonstrated that nanofertilizers can enhance plant growth and improve crop quality, uncertainty remains regarding their persistence in the environment and their potential impacts on surrounding ecosystems. It is still unclear whether nanofertilizers may enter food chains after application, accumulate across trophic levels, or ultimately pose risks to human health and higher-level consumers [104]. Such unresolved questions raise concerns about possible ecological disturbance, loss of biodiversity, and disruption of ecosystem balance. Evidence also indicates that excessive application rates can induce molecular-level alterations in plants and lead to nutrient imbalances [105]. Empirical studies further highlight these risks. The transfer of nano-TiO_2_ from Chironomus riparius larvae to Proisotoma minuta via biomagnification in a model food chain illustrates the potential for substantial nanoparticle fluxes into aquatic trophic networks originating from surface water environments [106]. In addition, nanoparticles may induce phytotoxicity through excessive generation of reactive oxygen species, resulting in oxidative stress, damage to cellular biomolecules, and interference with multiple biological pathways [107]. Given that nanoplastics are known to enter the human body through dermal contact, inhalation, or ingestion [108], nanofertilizers may exhibit similar exposure routes and associated health risks. For example, nano-TiO_2_ of different crystal phases has been widely detected in aquatic environments, where continuous industrial and commercial use contributes to its accumulation. Variations in crystal structure confer distinct physicochemical properties, environmental behaviors, and toxicity profiles toward aquatic organisms [109].

As a key direction in nanomaterial research, greater emphasis should be placed on elucidating the toxicity, biodegradability, and mobility of nanofertilizers, as well as their modes of action in plants, to ensure both ecological and human health protection. Alghofaili reported that magnesium-aluminum oxide nanofertilizers applied at elevated concentrations (≥200 mg L^−1^) significantly impaired multiple plant growth indicators, including germination rate, biomass accumulation, photosynthetic performance, and root and leaf elongation. At the cellular scale, pronounced damage was observed, characterized by membrane disruption, altered root morphology, and genotoxic effects [110]. To improve ecological safety evaluation, integrated approaches combining artificial intelligence and big data analytics should be employed to investigate nanofertilizer migration pathways within soil-water-crop systems, bioaccumulation patterns across trophic levels, and long-term ecological consequences. In parallel, standardized methods for assessing biodegradability are needed. Degradation experiments conducted under simulated field conditions would allow quantification of degradation kinetics and the toxicity of intermediate products throughout crop growth cycles, thereby providing essential data for ecological risk assessment.

Despite the potential advantages of nanofertilizers, their practical application remains constrained by high production costs and challenges associated with large-scale manufacturing. Reducing costs while enabling scalable and consistent production, therefore, remains a major technical barrier [111,112]. In addition, the tendency of nanomaterials to aggregate often necessitates pre-treatment or chemical modification to improve dispersibility during the formulation of slow- and controlled-release fertilizers. These additional processing steps increase system complexity and further hinder mass production [113].

In addition, global legal and regulatory systems governing the agricultural application of nanomaterials are still in their infancy. At present, legislation for agricultural nanomaterials (e.g., nano-pesticides, nanofertilizers) is still in the exploratory stage, with systemic shortcomings arising from insufficient technical understanding and limited regulatory experience. Within the European Union, nanomaterials fall under the regulatory scope of the Plant Protection Products Regulation (EC No. 1107/2009) and the current EU Fertilizing Products Regulation (EU) 2019/1009. Nano-enabled plant protection products are subject to case-by-case evaluation, typically requiring comprehensive environmental risk assessments prior to market authorization. These assessments generally emphasize toxicity to non-target organisms (e.g., pollinators and soil microorganisms) as well as information on environmental fate and degradation pathways. In the United States, the Environmental Protection Agency (EPA) regulates nano-pesticides under the Federal Insecticide, Fungicide, and Rodenticide Act (FIFRA). Nano-pesticides are evaluated individually, and registrants must demonstrate that nano-formulations are as safe as, or safer than, their conventional counterparts, supported by data on particle behavior, dispersibility, and potential bioaccumulation.

In the United States, nanoscale iron oxide has been approved for use as a food coloring agent. In Canada, the Canadian Environmental Protection Act (CEPA), which came into force in 1999, constitutes the principal federal framework governing the environmental and human health risks associated with nanotechnology-based products, including those intended for agricultural use [114]. Under CEPA, nanomaterials are evaluated and managed through the New Substances Notification Regulations, which provide a structured framework for risk assessment and regulatory oversight [115]. Taken together, these regulatory bodies pursue a common goal of safeguarding both the effectiveness and safety of nanotechnology-based agricultural products, while ensuring adherence to relevant regulatory requirements.

China developed basic nanomaterial classification standards (e.g., GB/T 39855-2021 [116]) and nanopesticide quality norms at an earlier stage. However, as of 2024, a comprehensive and updated safety assessment framework specifically addressing emerging nano-enabled agricultural inputs, such as nanofertilizers and nanopesticides, has not yet been formally established. China’s Ministry of Agriculture and Rural Affairs (MARA) has emphasized the need to develop safety standards for novel fertilizers and to revise pesticide registration systems, with increasing attention to the environmental risks associated with agricultural nanomaterials. Based on current academic and regulatory discussions, future regulatory efforts are likely to place greater emphasis on the migration and transformation of nanomaterials in soil–plant systems, as well as their potential impacts on groundwater and trophic transfer. Most developing countries have not yet established dedicated regulatory frameworks for agricultural nanomaterials and instead rely on existing pesticide and fertilizer regulations, which inadequately address nano-specific risks—such as nanomaterial-specific environmental fate and trophic transfer—thereby potentially creating substantial legal and regulatory barriers to their practical application [97,107]. Therefore, establishing and improving appropriate regulatory standards is essential, particularly through the integration of risk assessment and life cycle assessment frameworks, to systematically address nano-specific risks such as environmental fate, bioaccumulation, and trophic transfer [103,117].

### 5.2. Future Prospects

From the perspective of sustainable agriculture, the intrinsic physicochemical properties of nanomaterials provide the fundamental basis for the application of nanofertilizers, thereby establishing a close connection between nanomaterial technologies and sustainable agricultural development. By exploiting size-dependent and interfacial effects, nanomaterials are reshaping fertilizer application strategies within soil systems. First, nanofertilizers enable higher nutrient-use efficiency while reducing input requirements. Nano-carriers allow for precise regulation of nutrient release, leading to increases of approximately 10–20% in nitrogen and phosphorus utilization efficiency and reductions of more than 50% in nutrient losses. These improvements contribute to the mitigation of soil acidification and the eutrophication of aquatic environments. In practice, nanofertilizers can be applied through multiple approaches, including foliar spraying, seed coating, soil incorporation, and irrigation systems. Such flexible application modes support precision agriculture, improve nutrient management, facilitate soil restoration, and enable targeted correction of nutrient deficiencies [104,118].

Second, nanofertilizers can enhance soil fertility by improving soil physical and biological properties. Nano-clays and zeolite-based composite materials have been shown to optimize soil structure by reducing bulk density and increasing porosity, while simultaneously enhancing water and nutrient retention. In addition, these materials can stimulate microbial proliferation—for example, increasing actinomycete abundance by up to 72%, and promote soil enzyme activity and nutrient cycling efficiency. Third, nanofertilizers offer opportunities for targeted soil remediation while supporting plant growth. Certain materials, such as nano-hydroxyapatite, are capable of immobilizing heavy metals in soil, thereby reducing metal uptake by crops, including cadmium accumulation in rice. Similarly, layered silicate structures can adsorb and stabilize soil contaminants, providing additional pathways for ecological restoration in degraded agricultural soils.

Developing more environmentally friendly and low-cost nanomaterials will make nanofertilizers more competitive. Converting bio-organic waste into nano-agrochemicals is an economically viable and environmentally safe option. For instance, Sebastian synthesized potassium-doped nitrogen-based fertilizers using green chemistry, extracting nutrients from ripe banana peels to reduce resource waste. Chitosan nanoparticles, prepared via a modified ion gel method, were used as nutrient carriers for crops [119]. Zhu et al. employed KOH-activated persulfate to convert waste milk into humic acid-like acids and humic acid-like substances. They subsequently mixed the resultant product with attapulgite to prepare a slow-release nano humic acid-like fertilizer [120].

Advances in fabrication technologies can improve nanomaterial performance while lowering costs, making large-scale production more feasible. Metal nanoparticles (NPs) as nanofertilizers can help mitigate high production costs, excessive demand for pesticides and fertilizers, and soil degradation. When used as biosensors and seed priming agents, metal nanomaterials can also be integrated with precision agriculture to achieve accurate resource management [121].

Exploiting the multifunctionality of nanomaterials can allow them to serve dual roles as fertilizers and in other capacities, such as pesticides. By coupling zeolitic imidazolate framework-8 (ZIF-8) with RNA interference (RNAi), Huang et al. engineered a pH-responsive nano-agro-fertilizer composite designed for effective and synergistic suppression of Fusarium oxysporum wilt, while promoting crop growth, demonstrating broad application potential [122]. Li et al. engineered an antioxidant ferrous foliar fertilizer (ORFFF) using hollow silicon carriers at the micro/nano scale and incorporating in situ-formed vitamin C as the antioxidant component. This approach constitutes an antioxidant silicon-based nanosystem designed for the controlled foliar delivery of ferrous iron. Owing to its pH-responsive behavior, ORFFF allows the release rate of Fe(II) to be regulated over time, thereby accommodating the varying iron requirements of different crops and developmental stages [123].

Recent studies have also demonstrated the benefits of integrating machine learning (ML), artificial intelligence (AI), and artificial neural network (ANN)-based deep learning models with nano-engineered seed priming techniques. Such integration has been associated with improved seed diagnostic accuracy, more efficient crop management strategies, and the optimization of agricultural practices across multiple operational levels. By leveraging large-scale datasets together with real-time data processing, these computational frameworks enable data-driven decision-making related to planting schedules, harvest timing, and precision fertilization. The convergence of advanced data analytics with nanotechnology-enabled seed treatments, therefore, offers new opportunities to enhance agricultural sustainability through predictive modeling and adaptive resource allocation [124].

**Figure 9:**
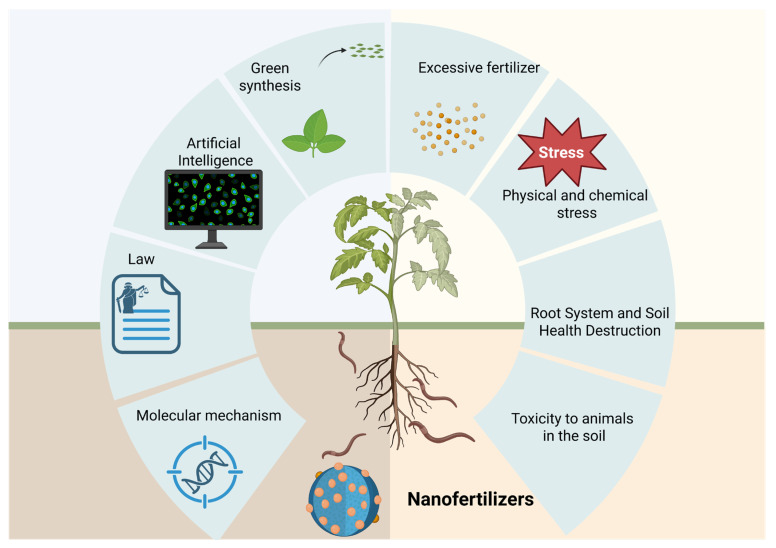
Nanofertilizers: Future Prospects for Sustainable Agriculture and Current Ecological Risks.

Through improvements in fertilizer use efficiency, reductions in environmental pollution, and enhancements in soil quality and beneficial microbial activity, nanomaterials can contribute to increased crop yield and improved crop quality, thereby providing strong support for the long-term development of sustainable agricultural systems (Figure 9).

## 6. Conclusions

This review demonstrates that the agronomic and ecological performance of nanofertilizers cannot be attributed solely to the inherent characteristics of nanomaterials. Instead, outcomes are strongly shaped by application rate, material composition, and the specific agronomic setting in which they are used. When applied at carefully controlled, low concentrations, well-designed nanofertilizers have the potential to increase nutrient utilization efficiency, improve soil physicochemical properties, and promote beneficial soil microbial processes. In contrast, studies employing excessive or agronomically unrealistic dosages frequently report phytotoxic effects and ecological disturbances, which likely account for the divergent findings observed across the literature.

At present, much of the available evidence is derived from short-term pot experiments conducted under simplified conditions and using non-representative application rates. Such experimental constraints reduce the robustness of current risk–benefit evaluations and limit their relevance to real agricultural systems. Addressing these gaps will require a shift toward field-based, multi-season studies that reflect practical management practices and allow for the assessment of long-term and cumulative impacts.

## Figures and Tables

**Table 1:** Core ecotoxicological effects of commonly used nanofertilizers.

Nanofertilizer Type	Application Dose Gradient	Affected Targets	Ecological Endpoint	Ecotoxicological Effect	Key References
Nano-ZnO	Low dose (<50 mg kg^−1^ soil); High dose (>200 mg/kg soil)	Soybean	Soil microorganisms (bacteria, fungi)	Low dose: No significant effects; High dose: Increased unstable Zn in microbial biomass; reduced bacterial and fungal CFUs; disrupted microbial community structure	[99,100]
Ag NPs	Low dose (<10 mg kg^−1^ soil); High dose (>50 mg/kg soil)	Soil microbial community	Soil microorganisms (Proteobacteria, Acidobacteria, ammonia-oxidizing bacteria/archaea); Fungal communities	Low dose: Enhanced microbial metabolic activity; increased Proteobacteria and Acidobacteria abundance High dose: Decreased amoA gene copy number; pronounced alteration of fungal community structure	[98]
Nano-biochar	Low dose (<1% *w*/*w*); High dose (>5% *w*/*w*)	Maize	Soil microorganisms (microbial biomass C/N); Maize nutrient uptake	Low dose: Maintained microbial function; improved nutrient cycling High dose: Reduced microbial biomass C and N; inhibited maize K uptake; microbial toxicity observed	[97]

## Data Availability

No new data were generated or analyzed in this study. All data discussed in this review are available in the cited literature and references therein.
